# Whiteside County Medical Society

**Published:** 1852-08

**Authors:** A. G. Porter

**Affiliations:** Secretary


### *Whiteside County Medical Society.*

On the 17th of June Whiteside County Medical Society held its annual session at Como, in the Odd Fellows' Hall, which had been kindly tendered them by the Order. Pres't A. Smith occupied the chair. A. T. Hudson, M.D., presented for membership Dr. J. W. Myers, who was duly elected. Treasurer's report was read, accepted, and placed on file. The President's Annual Address was then read before the Society, followed by the reports of cases and interesting remarks from some of the members.

The Society next proceeded to the election of officers for the ensuing year and until their successors are elected, which resulted as follows:

President—A. W. Benton, M.D., of Sterling; Vice President—A. T. Hudson, M.D., of Albany; Secretary—A. G. Porter, M. D., of Prophetstown; Treasurer—A. J. Grover, M.D., of Lyndon; Censors—Drs. Donaldson, Hudson, and Grover.

President then appointed H. C. Donaldson, M.D., of Como, to deliver an original essay on some subject connected with the profession, at the next semi-annual meeting.

On motion of Dr. Porter, Society adjourned to Albany, on the first Tuesday in December next, at 10 o'clock, A.M.

A. G. Porter, M.D., *Secretary.*

